# Indents

**Published:** 1920-05

**Authors:** 


### INDENTS

☞ **Brief Articles of Technical or Scientific Value will receive notice and comment. Contributions invited.**

RHEUMATIC FEVER FROM FOCAL INFECTION. Dr. Alexander Lambert has compiled statistics from Bellevue Hospital which indicate definite relation of dental infections to systemic conditions, such as tonsils and alveolar and peridental infections of a suppurative character. The article is well worth studying and appears in full in the *Journal of American Dental Association* for April 10, 1920. He says in part:

"These figures were obtained in gathering material for a report on health insurance to see whether or not there had been an actual diminution in the number of cases of acute rheumatic fever admitted to Bellevue Hospital, and to see whether any deductions could be drawn from them regarding the wisdom of including dental hygiene in the preventive measures of social insurance. They are too limited for any sweeping generalization. But it would seem that the actual and striking diminution of total admissions for rheumatic fever of the past two years was more than accidental, and that for this dental hygiene more than any other one factor was responsible. That tonsillectomy has also made its impression is most probable, as the diminution of both total number of admissions in the 20 to 29 year group and the percentage ratio to total number of this group is really most striking, especially since during this period of life the liability to rheumatic infection is especially noticeable. The average number admitted of 20 to 29 years of age during the four-year period 1911 to 1914 was 180, while during the five-year period 1915 to 1919 it was 110. The ratio to total admissions has also fallen from 17.8 per cent. for 1911 to 1914 to 15.3 per cent. for 1915 to 1919. The two periods from 20 to 29 are the periods in which the figures show most definitely an average reduction of percentage to total, combined with a definite reduction of total admissions. Have tonsillectomies begun to tell, or has it been oral and dental hygiene? Undoubtedly both, as ten years have not elapsed since tonsillectomy before 15 years of age has become widespread, so that it could not previously have affected the older years of this and other age groups in the community."

---

PREVENTABLE PYORRHEA. I am confident we should be impressed with the idea that pyorrhea is to be considered in the light of a preventable disease, and that it is up to the dental profession to prevent it. Most cases may be cured in the sense that we may arrest the disease; and that the disease may be arrested in the great majority of instances is beyond question. In this connection I see no difference between the anterior teeth and the posterior teeth—histologically and pathologically the disease is the same. It is true that work on the posterior teeth is more difficult than on the anterior, but with careful instrumentation most of these teeth can be saved. Pockets should be rendered self-cleansing, but that does not mean that they will need no further care; these teeth do need after care, it makes no difference whether they be treated surgically or otherwise. Those mouths need better prophylactic treatment than they have ever had before, or they are likely to suffer a recurrence. The mouth where roots have been denuded is more likely to accumulate deposits and needs prophylactic care on the part of both dentist and patient.

We do not get a reattachment of gum to the root surfaces, but suppuration may be arrested and its progress be retarded for years. After making my preliminary chart and marking a tooth "doubtful"—meaning that there was a doubt in my mind as to whether the tooth could be retained or not, by giving it the benefit of the doubt, and planing down the root surface, I have been surprised to find how many times the gum would settle down and become pink and healthy. It is often a good plan to mark these teeth "doubtful" and give them the benefit of the doubt, but if they do not clear up do not dismiss the patient finally until they have been extracted.—P. G. Puterbaugh, in *The Journal of the N. D. A.*

---

THE NEED OF OCCLUSAL RESTS. In all forms of removable bridgework or partial dentures, where clasps are used, some form of occlusal rest is necessary as a means of providing against subsequent settlement of the case. If this precaution is not observed, complete loss of occlusion and usefulness will soon follow.—Hart J. Goslee, D.D.S., in *Practical Hints.*

---

ENTERING DENTAL PRACTICE. My advice to young graduates is to get into practice for themselves as quickly as possible. A limited association with an established practitioner might be of advantage, but only if the older practitioner possess knowledge which he would be willing to impart. Above all considerations the association should be brief, unless indeed it should become permanent.—Dr. Ottolengui, in *Items of Interest.*

---

PRACTICING DENTISTRY AS A SPECIALTY. There is no suggestion here intended that the dentist should practice all the present specialties in dentistry. But he should be sufficiently versed in the problems, and particularly those problems of the other specialists which abut into his own field, that he shall be competent to advise with and even to work with the separate specialists.

Let us suppose that a new patient applies, possibly for the simple operation of refilling a tooth, where "a filling has dropped out." The operation involved may yield a fee of a few dollars. And too often the general practitioner of to-day fills that one tooth and dismisses the patient. In other words he permits the patient to diagnose his own case, and he treats only what the patient asks to have treated. Why not emulate the orthodontist and the prosthodontist? How? As follows:

Let us say that a casual examination of the teeth shows a number of poorly made and poorly finished amalgam and gold fillings; one or two gold crowns and two or three badly discolored teeth. The gums even in this cursory examination show turgidity in places.

The dental specialist should take from his cabinet the models of a well occluded set of teeth. With the aid of these models explain to the patient the mechanics of mastication; the importance of correct cusp formation and the uses of the sulci which serve as sluiceways for masticated food; the value of correct contact points in protecting the gingiva, and the rationale of having the interproximal spaces occupied by healthy gum tissue. Then point out with the aid of radiographs the diseased conditions that may exist without causing pain, and explain the dangers of systemic disease from focal infection. In other words, first prove to the patient that you yourself understand the value of a healthy effective masticating apparatus, and with this foundation it will not be difficult to persuade the patient to permit a thorough examination in order to make a thorough diagnosis. Perhaps at that very sitting radiographs could be taken of the suspected teeth at least. Then at the second sitting take good impressions and from these make as perfect models as are seen in the cabinets of the orthodontist. Finally, in the presence of the patient compare these models with the models of normal occlusion, and show where his previous fillings are at fault. Suggest the remedy and point out the value to his health of a complete restoration of his tooth forms and the resultant masticating efficiency.

Let us pause here to answer the comment of many readers, which we hear telepathically. Some who read this at this point will say, "Patients will not 'stand for it.' " If not, my friend, you are a poor salesman; worse yet, you are an incompetent dentist. Very seldom do physicians prescribe a course of treatment and have the patient decline to obey. But think of this. If a patient will pay thousands, literally thousands of dollars for bridged teeth, which at best are but poor substitutes for the natural organs, why will he not gladly pay for the complete restoration of his own teeth to their highest functioning activity? The answer, my dear colleague, is that he will! He will if you prove to him that it will be worth while.

Here is another point. The making of a bridge is usually an operation occupying a brief period, with a bill at the end of that period. The complete rearrangement, reorganization and restoration of a set of natural teeth, which had been marred by bad dentistry, is not necessarily a trying nor a financially burdensome task. It may be made to cover months or even two or three years of time. The study models having indicated the plan necessary, the most needful work should be done first, and the whole dental apparatus may then be slowly, but surely, improved by the removal of bad fillings and replacing them with better ones. In the end new models should be made and shown to the patient, together with those made at the outset, just as the orthodontist does. By this time the patient will have learned the value of the service in added comfort and improved health. From that time on he will need no urging to "go to the dentist." He can not be kept away. And he will tell his friends. He will tell them, "Don't have your teeth just filled. Have them thoroughly restored. Go to my dentist."—Dr. Ottolengui, in February *Dental Items of Interest.*

---

DENTAL PAIN. Dr. Edmund Kells contributes a valuable paper in the February *Dental Items of Interest* on various aspects of dental pains, some of which we think are very interesting.

Classification of dental pains he enumerates as follows: preoperative pain; reflex pain; operative pain; post-operative pain; mental pain. We don't quite understand why he makes no special class of the most important class of all, the pain of disease, whether reflex or referred. Under "preoperative pain" he suggests that it would be a good idea if all dentists would keep in mind how it is going to feel if the tooth he operates on is going to hurt as much as though it were his own tooth; then he will be careful and merciful. "Reflex pains" are often difficult to locate and treat. This requires knowledge in diagnosis as well as skill in treatment, in therapeutics or radical surgery. "Operative pains," painless operations, are generally a misnomer, but generally tolerable by some form of strategic instrumentation or therapeutic applications. It is inconsiderate to be inexcusable of one's patients, and is fatal as a business policy. Sympathetic encouragement is a most valuable and pain-relieving system to adopt in all methods of dental operations. "Post-operative pains": This requires the use of analgesics, anesthetics and anodynes, as well for the purpose of avoiding the pain incident to the operation as for the unavoidable pain. Post-operative pain has not been classified as a standardized treatment because there is such a diversity of causes. A large range of physical measures, complicated with therapeutic and surgical resources, are at command and the skillful application of them will best serve the thoughtful dentist. Such remedies as heat, cold, rest, emolients, narcotics, somnifacients, etc., all serve good purposes as indicated. "Mental pains": The attitude of mind of both patient and dentist needs to be most carefully guarded in all circumstances where sympathy for the patient and the most scientific and careful treatment are indicated. Patient and thoughtful consideration is of the utmost consequence in all cases, and it is many times the cause of utter failure because of carelessness or inhuman blundering. Mental pain may be very real and it should always be most carefully considered by the dentist.

---

SYMPTOMS ASSOCIATED WITH DENTITION. Dr. Ellsworth Moody cites the two following cases. They are not offered as proof of the contention that they are the result of teething in any sense of the word, but it is felt that the coincidence is very striking.

*Case I.* Male, age ten months. Brought to office because of cough. Physical examination absolutely negative except for swollen gums over the maxillary and mandibular second incisors. History of cough preceding eruption of the first incisors, which cleared up after their eruption. Blood count normal; urine negative; temperature 98.8 degrees F. by rectum. No medication was suggested, and after two days of careful physical examination and finding no other pathological reason for the cough the gums over the maxillary second incisors were scraped with a scalpel, allowing the teeth to come through. The cough disappeared immediately, to recur at the end of four days, when the mandibular second incisors were scraped through. The cough again stopped almost immediately.

*Case II.* Female, age nine months. Seen because of convulsions. Rectal temperature 103 degrees. General physical examination negative; reflexes normal; blood, spinal fluid and urine negative. Bulging of gums over nonerupted maxillary first incisors. A second convulsion came on during the examination. No treatment other than lancing gums. Within four hours the temperature had dropped; the child was apparently normal, and there were no more convulsions.

Moody has recently observed four cases whose history corresponds pretty closely to that of Case I. All cleared up within twenty-four hours after eruption of the teeth. In one case, elixir of terpin hydrate with heroin was given to an eleven months old babe, but did not affect the cough.

Lancing the gums apparently cured her.—*Medical Record and Cosmos.*

---

INDIRECT CASTING METHOD. I have derived more satisfaction out of the indirect method of casting, as taught by Dr. LeGro last summer, than from anything else I have learned in a long time. This method is especially fine for the casting of large inlays, restoring badly broken down molars and bicuspids or teeth from which you have removed gold crowns and wish to restore without crowning.

The method briefly described is as follows: Prepare the tooth as usual, removing all weak enamel walls and fit a copper band so it will cover all gingival margins. The band should be at least a half inch long. Remove band and fill with modeling compound, heat and force over the tooth, having the outer end of the compound quite hard so it can be used as a sort of a plunger to put pressure on the softer compound next to the teeth. Lay this aside.

Heat a small piece of inlay wax and press it into the cavity and allow the patient to bite into it. Place a piece of rubber dam over the wax and have patient bite again and grind on it. Chill and remove. The wax need not go to the gingival of the cavity at all.

Wrap a piece of paper around the modeling compound impression and reinforce with plaster. Mix amalgam very soft (sloppy) and make amalgam die, using mallet force to condense. Let set at least four hours—over night is better.

The wax being now thoroughly chilled, would probably break into a dozen pieces if you tried to place it on the model without warming it. This is accomplished by placing both the amalgam and the wax in water for a few minutes. The wax bite is then placed on the amalgam model and the excess carved away, preserving contact points and carving the occlusal surface according to the bite you have secured.

The wax is now removed and the amalgam die is oiled slightly, the wax replaced and the margins of the wax are all cut away and restored with new wax melted on the die with a spatula. Cut out but a small portion of the margin at a time and melt wax in the space and hold under pressure with a cold instrument while cooling.

After casting, the inlay is swaged on the amalgam die and polished, and if this technic is carefully carried out your contacts, occlusion and all should be just right and the inlay should require no fitting of any kind.—*C. M. Harris, D.M.D.*

---

Why a Professional Man Should Play Golf. By Charles Evans, Jr. There are many games perhaps better suited to boys and to very young men, but golf is the ideal game for the professional man. It is a pleasantly active and not too strenuous sport, and it is played outdoors, thus providing the much-needed fresh air and combining it with the right sort of exercise.

The lawyer in the crowded courtroom, the doctor in his office or beside a sickbed, the teacher in his classroom, all suffer from the lack of invigorating exercises out-of-doors; but of all classes of professional men it seems to me that the dentist stands the greatest need for outdoor recreation. His work is entirely indoors and hours of it are spent bending over patients. Some form of outdoor recreation would benefit not only the health of the man, but the quality of his work itself.

The game of golf seems to be the only one suited to the mature man. The sports of our boyhood and young manhood are much too lively for the man who is approaching or has passed thirty-five. I know that there was a time in my own life when I had to elect a major sport. I had played football and baseball not badly, and the football or baseball hero was the idol of every schoolboy.

The young crowd loves a "peppy sport," but the fact was brought home to me that these games belonged wholly to the days of our early youth, and that few men over thirty could play them, or even cared to. I do not mean to say that I ever loved another game as I do golf, but at times the attraction of the over-strenuous games reached me. The great hold that golf has upon us, however, grows out of its lasting quality. It is like an old and faithful friend walking beside us from youth to old age and to whom we can give as much or as little of our time as we desire.

The professional man can often spare a few hours a week for recreation, but if he can use them under an open sky with pleasant companionship, and an abounding interest in the assisted movements of a little white ball over fairways and putting greens in bunkers, or out of them, and all the thrilling adventures to be found between the first tee and the last hole, he is a fortunate man. One might travel far before finding such interesting changes as that elusive ball is capable of disclosing. You discover that your club and ball are a strangely interesting pair, and mind and body are so busily employed that all the troubles and ills of patients and clients slip easily away. And that is the great need of the professional man—something that makes him forget his work and gives him not too strenuous exercise in the open air.

---

A NATURAL GUM PINK. Many efforts are being made to esthelize the vulcanite denture. Some methods are better than others, many have weaknesses. I have found the following a desirable and quite satisfactory method: Take a strip one-eighth inch wide from a sheet of light, medium and dark pink, then a strip one-fourth of an inch wide from a sheet of Gould's gum pink, twist these into a rope and cut from it such pieces as you desire for packing. In some cases I find that a sixteenth of an inch strip of light orange may be added to the above with fine results. This method makes a stronger cast.—*F. W. F.*

